# Associations of meeting 24-h movement guidelines with stress and self-rated health among adults: is meeting more guidelines associated with greater benefits?

**DOI:** 10.1186/s12889-021-10979-3

**Published:** 2021-05-17

**Authors:** Kaja Kastelic, Željko Pedišić, Dean Lipovac, Nika Kastelic, Si-Tong Chen, Nejc Šarabon

**Affiliations:** 1 University of Primorska, Andrej Marušič Institute, Koper, Slovenia; 2 InnoRenew CoE, Izola, Slovenia; 3 Institute for Health and Sport, Victoria University, Melbourne, Australia; 4 Health Centre Murska Sobota, Murska Sobota, Slovenia; 5 University of Primorska, Faculty of Health Sciences, Izola, Slovenia

**Keywords:** Subjective health, Well-being, Time-use epidemiology, Daily Activity Behaviours Questionnaire

## Abstract

**Background:**

Several countries have recently issued 24-h movement guidelines that include quantitative recommendations for moderate-to-vigorous physical activity (MVPA), sedentary behaviour (SB), and sleep. This study explored the associations of meeting the 24-h movement guidelines with stress and self-rated health among adults, and whether the likelihood of favourable outcomes increases with the number of guidelines met.

**Methods:**

A total of 2476 adults aged 18 years and over completed a questionnaire on their time spent in MVPA, SB and sleep, frequency of stress (*never*, *very rarely*, *occasionally*, *often*, *every day*), self-rated health (*very good*, *good*, *fair*, *bad*, *very bad*), sociodemographic characteristics, and lifestyle variables.

**Results:**

In an ordinal logistic regression analysis adjusted for age, sex, body mass index, education, socio-economic status, employment, place of residence, living with or without partner, and smoking, lower odds of higher frequency of stress were found for those meeting the combined 24-h movement guidelines (adjusted odds ratio [OR] = 0.45; 95% confidence interval [CI]: 0.32, 0.63; *p* <  0.001), any combination of two guidelines (OR range: 0.48–0.63; *p* <  0.05 for all), and sleep guideline only (OR = 0.51; 95% CI: 0.35, 0.75; *p* = 0.001). Higher odds of better self-rated health were found for those meeting the combined 24-h movement guidelines (OR = 2.94; 95% CI: 2.07, 4.19; *p* <  0.001), combination of MVPA and SB guidelines (OR = 2.33; 95% CI: 1.57, 3.44; *p* <  0.001), combination of MVPA and sleep guidelines (OR = 1.78; 95% CI: 1.23, 2.59; *p* = 0.002), and MVPA guideline only (OR = 2.24; 95% CI: 1.50, 3.36; *p* <  0.001). Meeting more guidelines was associated with greater odds of favourable outcomes (*p* for linear trend < 0.001).

**Conclusion:**

Adults who meet the sleep guideline, any combination of two guidelines, or all three guidelines experience stress less frequently. Meeting the MVPA guideline alone or in combination with any other movement behaviour guideline was associated with better self-rated health. The likelihood of less frequent stress and better self-rated health increases with the number of guidelines met. Adults should be encouraged to meet as many movement behaviour guidelines as possible.

## Background

There has been a recent shift from studying the effects of sleep, sedentary behaviour (SB), and physical activity (i.e. movement behaviours) separately to simultaneously examining the effects of all movement behaviours that occur in a 24-h day [1–6]. This paradigm was motivated by the findings that sleep, SB, and physical activity are associated with health [7, 8], and that the amounts of time spent in these behaviours are perfectly collinear parts of the 24-h day – more time spent in one movement behaviour inevitably leads to less time spent in the remaining ones. The new paradigm has been widely accepted by researchers and international and national public health authorities. The World Health Organization and several countries have issued 24-h movement guidelines [9–18]. According to the novel 24-h movement guidelines, adults should, for example, limit their SB to no more than eight hours per day, accumulate at least 150 min of moderate-to-vigorous physical activity (MVPA) per week, and sleep seven to nine hours a day [12].

Studies among children and youth have suggested that adhering to recommendations for a higher number of individual movement behaviours (e.g. for 3 vs for 2, or for 2 vs for 1) is associated with greater health benefits [19, 20]. However, for adult populations, such evidence is scarce. Given that the 24-h movement guidelines for adults have recently been issued in several countries, from a public health perspective it is important to explore whether meeting more movement behaviour guidelines is associated with greater benefits. According to the framework for Viable Integrative Research in Time-Use Epidemiology (VIRTUE), such associations should be explored for a range of health outcomes [2]. For the purpose of this study, we selected stress and self-rated health as outcome variables, as they have often been explored in relation to movement behaviours.

Stress is commonly defined as a “physical, mental, or emotional strain or tension” [21]. It can be understood as individual’s response to events that they experience as threatening to their well-being or overwhelming [22]. Although it is a natural response, prolonged stress can have negative effects on health [23]. Chronic stress can substantially alter nervous, cardiovascular, endocrine, and immune functioning, and it is associated with various chronic diseases, including coronary heart disease, stroke, depression, and anxiety [24–28]. Frequent experience of stress is a pervasive issue: every third adult globally reports experiencing a lot of stress [29]. Furthermore, stress is associated with movement behaviours [30–32]. The relationship is considered to be bidirectional, with stress as a predictor of engagement in movement behaviours and engagement in movement behaviours as a predictor of stress. In particular, high stress seems to be associated with low levels of MVPA [32] and short sleep [31]. Findings on the association between SB and stress are inconsistent [33].

Self-rated health is one of the most widely used measures in epidemiological studies [34]. It represents one’s subjective evaluation of their own overall health status. Self-rated health is a strong predictor of mortality risk [35], and it was found to be consistent with the objective general health status [36]. Findings from the World Health Survey suggested that the global prevalence of poor self-rated health is around 10% [37]. Previous studies have found that high MVPA [38], low SB [39] and adequate sleep duration [40] are associated with better self-rated health. These relationships are also likely to be bidirectional [41].

Very few studies examined the associations of the 24-h movement behaviour composition with stress and self-rated health [7, 8, 42]. Previous studies that assessed combinations of movement behaviours in relation to stress have shown mixed findings. For example, Onodera et al. [43] found that reallocating time from SB to MVPA is associated with less stress. Oftedal et al. [44, 45] found a favourable association with stress for an overall ‘healthy’ combination of movement behaviours (i.e. sufficient MVPA, low SB, and sufficient sleep) and dietary habits. Some studies did not find a significant association between stress and movement behaviour compositions [46, 47]. Furthermore, studies on the association between movement behaviour composition and self-rated health suggested a positive role of MVPA [48] and light-intensity physical activity [47]. However, evidence on the association of adherence to the 24-h movement guidelines with self-rated health and stress is scarce.

Therefore, the aim of this study was to explore the associations of meeting the 24-h movement guidelines with self-rated health and the frequency of stress among adults aged 18 years and over. We focused on exploring whether the likelihood of favourable stress and self-rated health outcomes increases with the number of movement guidelines met. We hypothesised that meeting the combined 24-h movement guidelines (i.e. recommendations for sleep, SB, and MVPA) is associated with better self-rated health and lower frequency of experiencing stress. We also hypothesised that adhering to a higher number of individual recommendations is favourably associated with self-rated health and stress.

## Methods

### Data collection and participants

Data were collected among Slovenian residents aged 18 years and over (including young adults, middle-aged adults, and older adults) from November 2019 to March 2020 using an online survey. Participants were recruited via mailing lists, daily newspapers, web-portals, and social media. The participation in the survey was voluntary and anonymous. All participants provided informed consent before commencing with the survey. The study was performed in accordance with the Declaration of Helsinki and it was approved by the Republic of Slovenia National Medical Ethics Committee (approval number: 0120–557/2017/4).

A total of 2476 participants agreed to participate in the study and completed the survey. The survey data were cleaned based automated detection of participants who did not use web-based sliders for responding to specific questions (which indicated that they did not understand how to respond to these questions or did not want to respond). This led to exclusion of 55 participants. Additionally, 88 participants were excluded from this analysis, because they were under the age of 18 years (*n* = 5), they did not provide information on socio-demographic characteristics (*n* = 55), or they provided unrealistic responses on body weight (*n* = 3), and sleep time (*n* = 25). A higher proportion of excluded participants were less educated and unemployed than included participants. We found no significant differences between the excluded participants and the study sample in any other socio-demographic or lifestyle characteristic (data not shown). Data from 2333 participants were included in the final analysis.

### Measures

#### Movement behaviours

The amounts of time spent in MVPA, SB, and sleep were assessed using the Daily Activity Behaviours Questionnaire (DABQ) [49]. This 31-item questionnaire asks about MVPA and SB (in the work, transport, domestic, and leisure-time domains) and sleep in the past 7 days. According to Landis and Koch [50], the test-retest reliability of DABQ estimates is moderate (quadratic weighted Cohen’s kappa [*κ*_w_] = 0.58 for sleep, *κ*_w_ = 0.56 for SB, and *κ*_w_ = 0.47 for MVPA), the agreement of sleep estimates from DABQ and a time-use diary is substantial (*κ*_w_ = 0.66), and the agreement between DABQ and accelerometer-inclinometer estimates is moderate for SB (*κ*_w_ = 0.41) and fair for MVPA (*κ*_w_ = 0.32).

Based on their responses to DABQ, participants were categorised into the following eight groups: 1) meeting all 24-h movement guidelines; 2) meeting sleep and MVPA recommendations; 3) meeting sleep and SB recommendations; 4) meeting MVPA and SB recommendations; 5) meeting only sleep recommendation; 6) meeting only SB recommendation; 7) meeting only MVPA recommendation; and 8) not meeting any of the recommendations. The thresholds for meeting the recommendations were: at least 150 min of MVPA per week; less than eight hours of SB per day; and sleeping between seven and nine hours (7:00–9:59 h:mm) per day for adults and between seven and eight hours (7:00–8:59 h:mm) per day for older adults [12].

#### Outcomes

The outcome measures were self-rated health and frequency of stress. The question about stress was: “*How often do you feel tense, under stress, or great pressure?*” with the following response options: *never*, *very rarely*, *occasionally*, *often*, and *every day* [51]. The test-retest reliability of the stress frequency estimate is substantial (*κ*_w_ = 0.77) [50]. The question about self-rated health was: “*In general, how would you rate your current health status?*” with the following response options: *very good*, *good*, *fair*, *bad*, and *very bad*. The question has been validated before [36], showing a strong association with objectively assessed general health status, morbidity [52], and mortality [35] in the general population. Also, test-retest reliability was shown to be good-to-excellent [53].

#### Socio-demographic and lifestyle characteristics

We assessed age (continuous variable), sex, body mass index (BMI; calculated from self-reported body height and body weight, and categorised as *underweight or ‘normal’ weight* [< 25 kg/m^2^] / *overweight* [25.0 kg/m^2^ to 29.9 kg/m^2^] / *obese* [≥30 kg/m^2^]), level of education (using the question *“What is your highest completed level of education?”*, with the response options: *primary education, vocational secondary education, professional or general secondary education, short-term higher education*, and *professional or academic higher education* that were later grouped into *primary or secondary education* / *higher education*), self-perceived socio-economic status (using the question *“How would you rate your socio-economic status?”*, with the response options: *very high, high*, *middle*, *low,* and *very low* that were later grouped into *high or very high / middle / low or very low*), employment status (using the question *“Which option below best describe your working status/schedule?”*, with the response options: *employed in non-shift work*, *employed in shift work that includes only daytime shifts*, *employed in shift work that includes also night shifts*, and *unemployed* that were later grouped into *employed in non-shift work / employed in shift work / unemployed*), place of residence (using the question *“Do you live in urban or rural area?”*, with the response options: *urban area,* and *rural area*), living arrangement (using the question *“Do you reside with spouse/partner?”*, with the response options: *with partner,* and *without partner*) and smoking status (using the question *“Do you smoke or using oral tobacco?”*, with the response options: *yes – smoking every day, yes – smoking occasionally*, *no – never smoked,* and *no – quit smoking* that were later grouped into *not smoking / smoking*).

### Statistical analysis

The data were processed and analysed using R version 4.0.2 [54] and R Studio 1.3.959 [55] with the packages *dplyr* [56], *ggplot2* [57], *janitor* [58], *skimr* [59], *rstatix* [60], *MASS* [61], *brant* [62], and *generalhoslem* [63]. Absolute and relative frequencies (%) were calculated for all variables.

The associations of meeting the movement guidelines with stress and self-rated health were analysed using ordinal logistic regression (proportional odds) models with stress and self-rated health as outcome variables. The analyses were adjusted for all the above-mentioned socio-demographic and lifestyle variables and for self-rated health (in the analysis with stress as the outcome variable) or stress (in the analysis with self-rated health as the outcome variable). The adjustments for confounding were based on findings of previous studies [64–70].

To examine if the likelihood of favourable stress and self-rated health outcomes increases with the number of movement guidelines met, we ran additional regression models. In these models, the explanatory variable was an ordered factor denoting the number of movement guidelines met (i.e. none, one, two, or all three). We tested for linear and quadratic trends between the explanatory variable (the number of movement guidelines met) and the outcome (stress frequency or self-rated health). In all regression models, those who did not meet any of the guidelines were selected as the reference group.

Proportional odds assumption and goodness of fit for each of the ordinal logistic regression models were assessed using the Brant test [71], Hosmer–Lemeshow test, Lipsitz test, and Pulkstenis–Robinson tests [72]. The regression model with stress frequency as the outcome variable did not violate the proportional odds assumption, and the goodness of fit was acceptable, as indicated by the Brant test (*χ*^2^(69) = − 86.68, *p* = > 0.999), Lipsitz test (LR [9] = 11.98, *p* = 0.214), Hosmer–Lemeshow test (*χ*^2^(35) = 32.54, *p* = 0.588), and Pulkstenis–Robinson chi-square and deviance tests (*χ*^2^(7289) = 7222, *p* = 0.709; *D*^2^(7289) = 4272, *p* > 0.999). Similarly, the Brant test (*χ*^2^(69) = 66.84, *p* = 0.551), Lipsitz test (LR [9] = 4.96, *p* = 0.838), Hosmer–Lemeshow test (*χ*^2^(35) = 14.59, *p* = 0.999), and Pulkstenis–Robinson chi-square and deviance tests (*χ*^2^(7505) = 7036, *p* > 0.999; *D*^2^(7505) = 3592, *p* > 0.999) indicated that the regression model with self-rated health quality as the outcome variable did not violate the proportional odds assumption and that the goodness of fit was acceptable.

## Results

### Sample characteristics

The mean (± standard deviation) age of participants was 48 ± 14 years. Most participants were females and highly educated, had middle socio-economic status, and lived with their partner (Table 1). The participants’ responses on the question about frequency of stress ranged from *never* to *every day*, with approximately half of the participants (47%) reporting *occasionally* experiencing stress. The participants’ responses on the question about self-rated health ranged from *very bad* to *very good*, with approximately half of the participants (55%) rating their health as good. Only 25% of participants met all three guidelines, while 7% of participants did not meet any of the guidelines. Two guidelines were met by 41%, and a single guideline was met by 26% of participants.

**Table 1:** Participant characteristics

Characteristic	***n*** (%)
Age group	
18 to 44 years	896 (38)
45 to 64 years	1153 (49)
65 years or more	284 (12)
Female	1731 (74)
BMI	
Underweight or ‘normal’ (< 25 kg/m^2^)	1220 (52)
Overweight (25.0–29.9 kg/m^2^)	811 (35)
Obese (≥ 30 kg/m^2^)	302 (13)
Smoker	404 (17)
Education	
Primary or secondary education	687 (30)
Higher education	1646 (70)
Socio-economic status	
High or very high	277 (12)
Middle	1820 (78)
Low or very low	236 (10)
Living arrangement	
Living with partner	1785 (77)
Living without partner	548 (23)
Place of residence	
Urban	1219 (52)
Rural	1114 (48)
Self-rated health	
Very good	314 (13)
Good	1287 (55)
Fair	658 (28)
Bad	68 (3)
Very bad	6 (0.3)
Experiencing stress	
Every day	139 (6)
Often	689 (30)
Occasionally	1086 (47)
Very rarely	392 (17)
Never	27 (1)
Meeting guidelines	
None	171 (7)
Only for MVPA	221 (9)
Only for SB	175 (8)
Only for sleep	217 (9)
For SB and MVPA	279 (12)
For sleep and MVPA	370 (16)
For sleep and SB	309 (13)
For sleep, SB, and MVPA	591 (25)
Number of guidelines met	
None	171 (7)
1	613 (26)
2	958 (41)
3	591 (25)

Note: *BMI* body mass index; *SB* sedentary behaviour; *MVPA* moderate-to-vigorous physical activity

### Movement behaviours and stress

For the participants who only met the guideline for MVPA or for SB, we did not find a statistically significant difference from the reference group (i.e. those who did not meet any of the movement guidelines) in the frequency of stress (Table 2). Those who met the sleep guideline only had approximately two times greater odds of reporting lower frequency of stress, compared with the reference group. Similar associations with lower frequency of stress were found for meeting any two of the guidelines and for meeting the overall, combined guidelines.

**Table 2:** The association between meeting movement guidelines and the frequency of experiencing stress

	Odds Ratio [95% CI]	*p*
Guideline(s) met		
None	[*ref*]	
Only for MVPA	0.90 [0.61, 1.32]	0.582
Only for SB	0.75 [0.50, 1.12]	0.162
Only for sleep	0.51 [0.35, 0.75]	0.001**
For SB and MVPA	0.63 [0.43, 0.90]	0.013*
For sleep and MVPA	0.57 [0.40, 0.81]	0.002**
For sleep and SB	0.48 [0.33, 0.69]	< 0.001***
For sleep, SB, and MVPA	0.45 [0.32, 0.63]	< 0.001***
Number of guidelines met		
0	[*ref*]	
1	0.69 [0.50, 0.96]	0.027*
2	0.55 [0.40, 0.76]	< 0.001***
3	0.45 [0.32, 0.62]	< 0.001***
Linear trend		< 0.001***
Quadratic trend		0.428

Note: *CI* confidence interval; *SB* sedentary behaviour; *MVPA* moderate-to-vigorous physical activity. The regression models were adjusted for age, sex, body mass index, level of education, socio-economic status, employment status, place of residence (urban / rural), living arrangement (with partner / without partner), smoking status, and self-rated health. *p <  0.05, **p <  0.01, ***p <  0.001

In the regression model with the *number* of guidelines met as an explanatory variable, we found a significant linear trend. The likelihood of higher stress frequency decreased with the number of guidelines met.

### Movement behaviours and self-rated health

For the participants who only met the guideline for SB or for sleep, or for both sleep and SB, we did not find a statistically significant difference from the reference group (i.e. those who did not meet any of the movement guidelines) in self-rated health (Table 3). Those who met the MVPA guideline only had approximately two times greater odds of reporting better health, compared with the reference group. Similar associations with better self-rated health were found for meeting any two of the guidelines, except sleep and SB. Those who met the overall, combined guidelines had approximately three times greater odds of reporting better health, compared with the reference group.

**Table 3:** The association between meeting movement guidelines and self-rated health

	Odds Ratio [95% CI]	*p*
Guideline(s) met		
None	[*ref*]	
Only for MVPA	2.24 [1.50, 3.36]	< 0.001***
Only for SB	1.43 [0.94, 2.19]	0.099
Only for sleep	1.02 [0.68, 1.53]	0.919
For SB and MVPA	2.33 [1.57, 3.44]	< 0.001***
For sleep and MVPA	1.78 [1.23, 2.59]	0.002**
For sleep and SB	1.37 [0.94, 2.00]	0.100
For sleep, SB, and MVPA	2.94 [2.07, 4.19]	< 0.001***
Number of guidelines met		
0	[*ref*]	
1	1.49 [1.06, 2.10]	0.022*
2	1.76 [1.26, 2.45]	0.001**
3	2.92 [2.05, 4.15]	< 0.001***
Linear trend		< 0.001***
Quadratic trend		0.599

Note: *CI* confidence interval; *SB* sedentary behaviour; *MVPA* moderate-to-vigorous physical activity; *BMI* body mass index. The regression models were adjusted for age, sex, body mass index, level of education, socio-economic status, employment status, place of residence (urban / rural), living arrangement (with partner / without partner), smoking status, and frequency of stress. *p < 0.05, **p < 0.01, ***p < 0.001

In the regression model with the *number* of guidelines met as an explanatory variable, we found a significant linear trend. The likelihood of better self-rated health increased with the number of guidelines met.

## Discussion

### Main findings

The results of this study suggest that meeting the sleep guideline only, any combination of two movement behaviour guidelines, or all three movement behaviour guidelines is associated with less frequent stress. Meeting the MVPA guideline alone or in combination with any other movement behaviour guideline is associated with better self-rated health. We did not find significant associations with stress frequency and self-rated health for meeting the SB guideline only. The likelihood of less frequent stress and better self-rated health increases with the number of guidelines met. These findings highlight the importance of encouraging adults to meet as many movement guidelines as possible, while meeting the sleep guideline seems to be particularly important for coping with stress and meeting the MVPA guideline seems to be particularly important for improving self-rated health.

### Comparison with previous studies

Our findings are in accordance with the results of two studies conducted by Oftedal et al. [44, 45], in which the latent class representing a favourable movement behaviour composition (i.e. sufficient MVPA, low SB, and sufficient sleep) was associated with the lowest likelihood of mental distress, compared with the remaining latent classes that represented unfavourable movement behaviour compositions. These comparisons should, however, be taken with caution, because the latent classes in the Oftedal et al. [44, 45] studies were based not only on movement behaviours but also on dietary habits. Furthermore, two other previous studies [46, 47] did not find a significant association between movement behaviours and stress, possibly because of their significantly smaller sample sizes compared to the current study. In our study, the strength of the association with stress frequency was almost identical across the groups meeting a combination of any two guidelines. It might be that for coping with stress the number of movement behaviour guidelines met matters more than which specific combination of movement guidelines is met.

Within the composition of movement behaviours, MVPA often shows the strongest positive association with mental well-being [73–76]. However, we did not find a significant association between meeting the MVPA guideline and stress frequency. Leisure-time MVPA is often promoted as a strategy to manage stress [77, 78]. It might be that some individuals in our sample met the MVPA guideline in the attempt to cope with frequent stress. Such cases could have attenuated the inverse association between MVPA and stress frequency in our sample, but this has to be confirmed in future, longitudinal studies.

Our findings regarding self-rated health are in accordance with the results of Oftedal et al. [44] study, in which all three latent classes representing unfavourable movement behaviour compositions had significantly worse self-rated health than the latent class representing a favourable composition of movement behaviours. In the Oftedal et al. [44] study, the poorest self-rated health status was found among those with a combination of low PA, high SB, and inadequate sleep, which is also congruent with the results of our study. As mentioned earlier, these comparisons should be taken with caution, because the latent classes in the Oftedal et al. [44] study were based not only on movement behaviours but also on dietary habits.

Our study showed that meeting the MVPA guideline alone or in a combination with meeting any other movement guideline is associated with better self-rated health, which is in accordance with previous findings. The positive association of MVPA with self-rated health among adults (while considering other movement behaviours) was also found in the von Rosen and Hagströmer [48] study. Similarly, results of the Park et al. [47] study suggested that increasing the time spent in MVPA at the expense of the time spent in SB is associated with better self-rated health in adults. Within the composition of movement behaviours, MVPA often shows the strongest positive association with health [1, 7, 42, 73]. The findings in our sample seem to be in line with these findings.

Similar as in our sample of adults, meeting more movement behaviour guidelines was found to be associated with better health and well-being among Australian adolescents [79]. These findings have important implications for health promotion, as they highlight the importance of encouraging adults to meet as many movement behaviour guidelines as possible. Promoting the integrated 24-h movement guidelines might be a feasible way to do it [12, 16–18].

### Practical implications

Given the high prevalence of experiencing stress and poor self-rated health in the population, the relatively large reductions in odds of these outcomes associated with meeting the 24-h movement guidelines can be considered practically meaningful from the public health perspective. Public health interventions and strategies encouraging people to engage in 150 min or more of MVPA per week, sit less than 8 h per day, and sleep between 7 and 9 h per day are likely to be good investments in reducing the frequency of stress and improving self-rated health among adults.

### Strengths and limitations

The key strengths of the current study include a large sample size and exploring how meeting recommendations for specific movement behaviours (and all their combinations) is associated with two important health outcomes. Several limitations of the study should also be highlighted. First, the study sample was not fully representative of the general population (for example, in terms of distributions of sexes, education levels, and smoking status), which limits the generalisability of the findings. However, the sample was representative of the Slovenian population in terms of meeting the MVPA guideline, stress, self-rated health, age, BMI, socio-economic status, place of residence, and living arrangement [80]. Second, given that the data were collected using self-reports, the findings of the study may have been affected by recall errors and social desirability bias. Third, we did not consider guidelines on screen time and muscle-strengthening activity [12]. Also, given that the 24-h movement guidelines for adults do not include specific recommendations for different domains in which MVPA and SB can take place (e.g. work, transport, domestic, leisure time), we did not consider possible differential outcomes of domain-specific activities. Finally, no conclusions about causality could be drawn, because the study was cross-sectional. Studies with longitudinal and experimental designs are warranted to better understand the causal relationships.

## Conclusion

Our results suggest that adults aged 18 years and over who meet the sleep guideline, any combination of two movement behaviour guidelines, or all three movement behaviour guidelines experience stress less frequently, compared with those who do not meet any of the guidelines. Meeting the MVPA guideline alone or in combination with any other movement behaviour guideline is associated with better self-rated health. The likelihood of less frequent stress and better self-rated health increases with the number of guidelines met. These findings highlight the public health importance of encouraging adults to meet as many movement behaviour guidelines as possible, while meeting the sleep guideline seems to be particularly important for coping with stress and meeting the MVPA guideline seems to be particularly important for improving self-rated health.

## Data Availability

The datasets used and/or analysed in the current study are available from the corresponding author upon reasonable request.

## Abbreviations

BMI: Body mass index
CI: Confidence interval
DABQ: Daily Activity Behaviours Questionnaire
MVPA: Moderate-to-vigorous physical activity
OR: Odds ratio
SB: Sedentary behaviour
VIRTUE: Viable Integrative Research in Time-Use Epidemiology
